# Supplementary material for: Patterns of Intron Gain and Loss in Fungi
Source: PLoS Biol. 2004 Nov 30;2(12):e422. doi: 10.1371/journal.pbio.0020422 (PMC532390; doi:10.1371/journal.pbio.0020422)
Supplement: Table S1 — Also available at http://genes.mit.edu/NielsenEtAl/. (4.3 MB ZIP). [file pbio.0020422.st001.zip › NielsenEtAl/html/534.html]

AN5442.1.NCU00477.1.MG05663.1.FG06895.1


```
 CLUSTAL W (1.82) Multiple Sequence Alignments - Introns Inserted


Sequence 1: NCU00477.1	554 aa
Sequence 2: MG05663.1	552 aa
Sequence 3: FG06895.1	540 aa
Sequence 4: AN5442.1	552 aa
Alignment Length: 564 aa
Number Identitical Residues: 313 aa
Alignment Score (without introns) 15159


MG05663.1 	MRIATSTLLVGAASAAFAP--QDGTQRVLNG-----FDSIKSAAHSIQKP-LQTFEEAMA
NCU00477.1	MRISASTVLLGAASAASAASFQNQAQHVLADNFHKAHDAVKPVADSFAHTTLESFEEAFN
FG06895.1 	MRLSTSALVLGAASSAMGL-----EQQILGG-------DSKPLIDLDVNAWTKPLEKFFG
AN5442.1  	MRVLPATLLVGAATAATPA------QQVLGG-LQDFGNAVQDAMHENLPKINKPLEAFQE
          	**: .:::::***::*         *::* .   .  .  :   .       :.:*    

MG05663.1 	SMTS--EAKANWDHLKLLVPDAEEQAKTFFPKKPKPASRKPDSAWDHIVKGADIQAMWVE
NCU00477.1	GMNS--QAKALWDEIKLLVPENAFDKPTWFSK-PKAAKRRKD--WDHVVKGADVQKLWVK
FG06895.1 	EISS--EAKAVWDEVTLLAPDAVEAFKKQVLTEPKKVNRRPDSHWDHVVKGADVQSLWTE
AN5442.1  	QLKSLYEAREFWEEVANAFPQNLDHNP--VFSLPKKHTRRPDSHWDHIVRGADVQSVWVT
          	 :.*  :*:  *:.:    *:        . . **  .*: *: ***:*:***:* :*. 

MG05663.1 	GATPEETHRKIDGKLDNYNLRARSVDPSKLGVD-TVKQYSGYLDDEANDKHLFYW1FFES
NCU00477.1	GAD-GEKHREVGGQLDNFNLRVKSVDPSKLGVD-KVKQYSGYLDDEENDKHLFYW1FFES
FG06895.1 	KNG--EKHREVGGKLENYNLRAKKVDPSKLGVD-KVKQYSGYLDDEEEDKHLFYW1FFES
AN5442.1  	GEN-GEKEREIEGKLEAYDLRIKKTDPSSLGIDPDVKQYTGYLDDNENDKHLFYW1FFES
          	     *..*:: *:*: ::** :..***.**:*. ****:*****: :******* ****

MG05663.1 	RNDPKNDPVVLWLNGGPGCSSLTGLLFELGPGAINAKIEIVHNPYAWNNNASVIFLDQPV
NCU00477.1	RNDPKNDPVVLWLNGGPGCSSLTGLFLELGPSSIDKKLRVVSNEYAWNNNASVIFLDQPV
FG06895.1 	RNDPKNDPVVLWLNGGPGCSSLTGLFLELGPASVNKKIEIVNNPESWNNNASVIFLDQPV
AN5442.1  	RNDPKNDPVVLWLNGGPGCSSLTGLFMELGPSSIDENIKPVYNPYAWNSNASVIFLDQPV
          	*************************::****.::: ::. * *  :**.***********

MG05663.1 	NVGYSYSGGSVSNTVAAGKDIYALLTLFFHQFPEYAKQDFHIAGESYAGHYIPVFASEIL
NCU00477.1	NVGYSYSGNAVSNTVAAGKDVYALLTLFFHQFPEYAKQDFHIAGESYAGHYIPVFASEIL
FG06895.1 	NVGYSYSGGSVSNTVAAGKDIYALLTLFFHQFPEYAKQDFHIAGESYAGHYIPVFANEIL
AN5442.1  	NVGYSYSGSTVSDTVAAGKDVYALLTLFFKQFPEYAEQDFHIAGESYAGHYIPVFTSEIL
          	********.:**:*******:********:******:******************:.***

MG05663.1 	SHKKRNINLKSVLIGNGLTDGLTQYEYYRPMACGEGGWKAVLSESECQAMDNALPRCQSM
NCU00477.1	SHKDRNINLKSVLIGNGLTDPLTQYEHYRPMACGEGGYPAVLSESECRSMDNALPRCQSL
FG06895.1 	SHDDRNINLKSVLIGNGLTDGYTQYAYYRPMACGDGGYPSVLSESECQSMDNALPRCQSL
AN5442.1  	SHQKRNINLKSVLIGNGLTDGLTQYEYYRPMACGEGGYPAVLDESSCRSMDNALGRCQSM
          	**..****************  *** :*******:**: :**.**.*::***** ****:

MG05663.1 	IQNCYDSGSVWSCVPASIYCNNAMIGPYQRTGRNVYDIRGPCKDSGNLCYPELGYISEYL
NCU00477.1	IRNCYESGSVWSCVPAAIYCNNQFIGPYQRTGQNVYDIRGKCEDDSNLCYSALGWISDYL
FG06895.1 	IKGCYESGSAWSCVPASIYCNNAMMGPYQRTGQNVYDIRGKCEDGNNLCYSGLGYIADYL
AN5442.1  	IESCYNSESAWVCVPASIYCNNALLAPYQRTGQNVYDVRGKCEDESNLCYKGMGYVSEYL
          	*..**:* *.* ****:***** ::.******:****:** *:* .****  :*::::**

MG05663.1 	NRREVMEALGAEVSSYDSCNFDINRNFLFQGDWMQPYHRLVPELLNQIPVLIYAGDADFI
NCU00477.1	NQKDVMDALGVEVEGYESCNFDINRNFLFQGDWMQPFHRLVPGILKEIPVLIYAGDADFI
FG06895.1 	NREEVKEALGAEVSSYDSCNMDINRNFLFAGDWMQPYHQLVPNVLDKIPVLIYAGDADFI
AN5442.1  	NKPEVRAAVGAEVDGYDSCNFDINRNFLFHGDWMKPYHRLVPGILEQIPVLIYAGDADFI
          	*: :*  *:*.**..*:***:******** ****:*:*:*** :*.:*************

MG05663.1 	CNWLGNQGWTEALEWKGKKDYNRADYSPLTLASAHDV-KPYGKVKSSGNFTFMKIFEAGH
NCU00477.1	CNWLGNKAWSEALEWPGKNGFNKAELEDLSLPKAD---KEYGKVKSSGNFTFMQIYQAGH
FG06895.1 	CNWLGNQAWTDKLQWSGQKDFSHADLKP--LKHAG---KEYGKVKSSGNFTFMQIYGAGH
AN5442.1  	CNWLGNKAWTEALEWPGHKEFAAAPMEDLKIVDNEHTGKKIGQIKTHGNFTFMRLYGGGH
          	******:.*:: *:* *:: :  *  .  .:      .*  *::*: ******::: .**

MG05663.1 	MVPYDQAEPSVDFVNRWLAGEWFAA
NCU00477.1	MVPMDQPENSLDFLNRWLGGEWFEQ
FG06895.1 	MVPMDQPEASSDFFNRWLSGEWF--
AN5442.1  	MVPMDQPEASLEFFNRWLGGEWF--
          	*** **.* * :*.****.****
```
